# Dietary Supplement Use by Community-living Population in Japan: Data from the National Institute for Longevity Sciences Longitudinal Study of Aging (NILS-LSA)

**DOI:** 10.2188/jea.16.249

**Published:** 2006-11-03

**Authors:** Tomoko Imai, Mieko Nakamura, Fujiko Ando, Hiroshi Shimokata

**Affiliations:** 1 Department of Epidemiology, National Institute for Longevity Sciences, Japan.

**Keywords:** Dietary Supplements, Nutrition Surveys, Cohort Studies, Minerals, Vitamins

## Abstract

**BACKGROUND:**

There are few studies about dietary supplement use and nutrient intake from these products in Japan. The purpose of this study was to clarify (1) the prevalence of dietary supplement use, (2) the characteristics of dietary supplement users, (3) nutrient intake from dietary supplements, and (4) the existence of dietary supplement users who took excessive nutrients from these products.

**METHODS:**

To collect the information on dietary supplement use in the previous year and nutrient intake from these products, we conducted a self-administered dietary supplement frequency questionnaire. The subjects were 2,259 people aged 40-82 years. Dietary supplements were grouped into 8 major categories. A dietary supplement database was developed to estimate nutrient intake from these products. Excess users were defined as people who consumed more nutrient than the tolerable upper intake level of the Dietary Reference Intakes for Japanese.

**RESULTS:**

In the previous year, 55 % of males and 61 % of females consumed dietary supplements. Dietary supplement use was especially prevalent in females, subjects who felt unhealthy, and subjects who were more careful of maintaining an appropriate weight, though the association was affected by the frequency of dietary supplement use. The most common dietary supplements were drink type in males and vitamins in females. Some nutrient values obtained from dietary supplements were higher than those from food. Excess users were found for intake of vitamin A, B_6_, K, niacin, iron, and magnesium.

**CONCLUSIONS:**

It is important to clarify dietary supplement use and to estimate nutrient intake from these products.

Because sales of dietary supplements have increased in Japan,^1^ it is conceivable that striking growth in the use of dietary supplements will occur in Japan, as it has in the USA and other developed countries. Assessing nutrient intake from dietary supplements, especially micronutrient intake, is very important. Because the levels of some micronutrients contained in these products are much higher than those contained in food,^2^^-^^4^ people can easily consume such nutrients at toxic levels.^5^^-^^9^ To monitor nutrient intake from dietary supplements is an important issue for public health. Furthermore, to assess nutrient intake from dietary supplements is essential for the development of nutritional epidemiologic studies. Lack of inclusion of dietary supplements in nutrient intake could possibly cause misclassification of individuals with regard to their total nutrient intake.^2^^-^^4^^,^^10^^,^^11^ However, there have been very few studies on dietary supplement use in Japan. There is still uncertainty about the prevalence of dietary supplement use, nutrient intake from these products, and existence of users who consume extremely high levels of nutrients. One reason for the delay in the study of dietary supplement use in Japan might be due to the lack of a dietary supplement database. An extensive database that includes nutrient contents of dietary supplements is necessary for evaluating nutrient intake from these products; however, such a database has not been established or distributed in Japan. In contrast, several studies have attempted to estimate quantitatively the amount of nutrient intake from these products in the United States and European countries.^2^^-^^11^

Therefore, we conducted a self-administered dietary supplement frequency questionnaire to collect information on dietary supplement use in the National Institute for Longevity Sciences-Longitudinal Study of Aging (NILS-LSA), and developed a database of dietary supplements to estimate the amount of nutrient intake from these products. The purpose of this study was to clarify the following four points: (1) the prevalence of dietary supplement use, (2) the characteristics of dietary supplement users, (3) nutrient intake from dietary supplements, and (4) the existence of dietary supplement users who took excessive nutrients from these products.

## METHODS

### Subjects

The subjects were 2,259 males and females aged 40 to 82 years who participated in the second wave examination of the NILS-LSA (from April 2000 through May, 2002). The NILS-LSA is a comprehensive population-based longitudinal study of aging, which started in 1997. The participants were stratified by both age and sex, and were randomly selected from resident registrations in the city of Obu and town of Higashiura in central Japan. The numbers of males and females recruited were similar and the baseline age was 40 to 79 years, with the similar numbers of participants in each decade of age (40s, 50s, 60s, 70s). At the first wave examination, we sent an invitation letter to 7,790 people and 3,434 people replied. A total of 2,267 people participated in the first wave examination. All participants gave their informed consent before they participated in the study. Details of the study purpose, design, and examination procedures have been described elsewhere.^12^

### Definition and Categories of Dietary Supplements in the NILS-LSA

Dietary supplements were defined as supplements to meals containing any dietary ingredients from unnatural food forms such as capsules, tablets, powders, or liquid. Dietary supplements included vitamins, minerals, herbs, botanical products, and other substances (e.g., enzymes, organ tissues, metabolites, concentrates, and constituent extracts of these substances). Dietary supplements also included prescription medicine and non-prescription medicine, but functional foods and modified foods were not included in the category of dietary supplements.

Dietary supplements were grouped into eight major categories on the basis of primary nutrient content or similarity in overall ingredients and rationale for use.^13^ In addition, we defined “drink type” separately, because Hakura et al^14^ reported that “drink type” dietary supplements were widely consumed in Japan. The major categories of dietary supplements used in the NILS-LSA were (1) vitamin, (2) mineral, (3) fatty acid, (4) amino acid, (5) dietary fiber, (6) drink type (liquid type dietary supplement for recovery from tiredness, health promotion, etc., with a serving size of about 30 mL to 200 mL. The drink type category included quasi-drugs, but did not include beverages), (7) medicine (prescription and non-prescription medicines which contained some nutrients, except medicines which were classified into categories 1 to 6, e.g. cold remedies with vitamin C), and (8) others (These formulations included compounds that did not fit into any other category and were not described in the Standard Tables of Food Composition in Japan, Fifth Revised Edition,^15^ for example, flavonoids, carotenoids expect beta-carotene, propolis, and so on) (Table 1). In addition, vitamins, minerals, and fatty acids were further classified into sub-categories. We use the term “all” dietary supplements which consisted of eight categories, when we did not consider the categories of the dietary supplements.

**Table 1.** Categorization of dietary supplements by the National Institute for Longevity Sciences Longitudinal Study for Aging.

Category	Description or sub-category
1. Vitamin*	14 sub-categories
	Multivitamin, Vitamin A, Vitamin D, Vitamin E, Vitamin K, Vitamin B_1_, Vitamin B_2_, Vitamin B_6_, Vitamin B_12_, Niacin, Vitamin C, Folic acid, Biotin, and Pantothenic acid

2. Mineral*	4 sub-categories
	Calcium, Iron, Magnesium, and Other minerals

3. Fatty acid*	6 sub-categories
	Linoleic acid, Linolenic acid, Stearic acid, Docosahexaenoic acid, Eicosapentaenoic acid, and Other fatty acids

4. Amino acid	Formulations containing mainly of single amino acids and some proteins

5. Dietary fiber	Water soluble and water insoluble dietary fibers

6. Drink type	Liquid type dietary supplement for recovery from tiredness, or health promotion, etc.
	The amount consumed at one time is about 30 mL to 200 mL. Includes quasi-drugs and medicinal drugs but does not include beverages.

7. Medicine	Prescription and non-prescription medicines which contain some nutrients, except medicines which are classified into categories 1 to 6. Example : remedies for cold which contain vitamin C.

8. Others	These formulations included compounds that do not fit into any other category. Example: flavonoids, carotenoids other than beta-carotene, catechin, and herbal products (propolis, royal jelly, chlorella, garlic, etc.)

Dietary supplements were defined as supplements to meals including any dietary ingredients from unnatural food forms such as capsules, tablets, powders, or liquids. Dietary supplements included prescription medicine, and non-prescription medicine, but functional foods and modified foods were not included in the category of dietary supplement.

*: Further classified into sub-categories shown in the Table.

### Assessment of Dietary Supplement Intake

A self-administered questionnaire was used to assess dietary supplement intake. First, it was mailed to the participants and the participants were asked to record it by themselves at home before the study examination. Then, the participants came to our center to get the study examination. At the examination, the questionnaire was reviewed by trained dietitians through an interview that took approximately 10 minutes. In the questionnaire, participants were asked whether they had taken any dietary supplement in the previous year. In case they had taken any dietary supplement, the name of the product, manufacturer or distributor, serving size and frequency of intake in the previous year (6 categories, i.e., less than once per week, 1-2 times per week, 3-6 times per week, one per day, 2 times per day, and 3 or more times per day) were also recorded.

### Definition of Dietary Supplement Users

Dietary supplement users in the present study were defined as persons who took any dietary supplement at least once in the previous year. Users of dietary supplements were categorized into three groups: “daily users”: those who reported any dietary supplement use once a day or more for the past 12 months, “weekly users”: those who reported any dietary supplement use once a week or more but less than once a day for the past 12 months, and “seldom users”: those who reported any dietary supplement use once a year or more but less than once a week for the past 12 months. When a participant had taken multiple dietary supplements in a major category or in a sub-category, the user category was defined based on the dietary supplement with the highest frequency of use. We used the term “any users” when we did not consider the frequency of use. “Weekly users” and “daily users” were considered to be “regular users”. “Seldom users” were excluded when we calculated the amount of nutrient intake from dietary supplements.

### Development of Dietary Supplement Database

A new dietary supplement database was developed for the NILS-LSA based on information obtained from the study participants and additional intensive investigation. We asked dietary supplement users to bring the products to the study visit. Then, the labels of the products were transcribed or photocopied to get information on the nutrient contents. In case dietary supplement users did not bring the products or could not provide enough information about the products at the visit, we asked them to send the labels of the products by mail. In addition, when information on nutrient content was not available from users, we tried to get it directly from the manufacturer or distributor of the products. We created a database of dietary supplements that included the names of products, manufacturer and/or distributor and nutrient contents in standardized units such as a tablet or a capsule.

Some products in which nutrient content was not described were excluded when we developed the database, and we did not calculate the nutrient intake from these products (62 products). Finally, we succeeded in constructing a database of 902 dietary supplement products in May 2002.

### Assessment of Nutrient Intake from Dietary Supplements

Energy and nutrient intake from “all” dietary supplements among “regular users” was estimated using the frequency, amount of intake and nutrient contents in the dietary supplement database. The frequency of dietary supplement intake per day was quantified during the calculation (0.2 for 1-2 per week, 0.6 for 3-6 per week). If a participant reported uncertainty about the information on dietary supplement intake, that dietary supplement was excluded from the calculation of nutrient intake (7 males and 22 females were excluded from the analysis because they reported uncertainty about the information on dietary supplement intake). When a participant had taken various kinds of dietary supplements, energy and nutrient intake were summed across all dietary supplements. Nutrient intakes from “all” dietary supplements were compared with those from food according to the results of the National Nutrition Survey in Japan 2002.^16^

Participants who daily consumed some nutrients at more than the tolerable upper intake level (UL) in the 6th Edition^17^ or 2005 Edition^18^ of Nutrient-Based Dietary Reference Intakes (DRIs) in Japan were defined as “excess users”. The ULs for adults in the 6th Edition of DRIs were as follows: 5,000 IU for vitamin A, 2,000 IU for vitamin D, 600 mg *α*-TE for vitamin E, 30,000*μ*g for vitamin K, 30 mgNE for niacin, 100 mg for vitamin B_6_, 2,500 mg for calcium (under 70 years old), 40 mg for iron, and 650 mg (50 years old and over) or 700 mg (40 to 49 years old) for magnesium. The UL for adults in the 2005 Edition of DRIs were as follows: 3,000*μ*gRE (10,000 IU) for vitamin A, 50*μ*g (2,000 IU) for vitamin D, 600 mg (70 years old and over for females) to 800 mg (40 to 69 years old for males) for vitamin E, 300 mgNE for niacin (the amount of mg of nicotinic acid amide was used), 60 mg for vitamin B_6_, 2,300 mg for calcium, and 40 mg (40-49 and 70 years old and over for females) to 55 mg (40 to 49 years old for males) for iron.

### Other Variables

Sociodemographic and lifestyle characteristic data, such as smoking habits, subjective health status, total family annual income, education, marriage status, and care of maintaining appropriate weight, were collected using a questionnaire. The body mass index (BMI) was calculated using the formula (weight (kg)/height (m)^2^). Energy intake from food, energy intake from fat, and total alcohol intake were assessed through 3-day weighed dietary records (3DR). 3DR was carried out on three continuous days (two weekdays and one weekend day). The average intakes of nutrient per day were calculated according to the 5th Edition Standard Tables of Foods Consumption and other resources.^19^

### Statistical Analysis

The prevalences of “all” dietary supplement users among males and females were compared by the chi-squared test by user category (any, seldom, weekly, and daily). Sociodemographic and lifestyle characteristics of “all” dietary supplement users and nonusers were compared by the Cocran-Mantel-Haenszel test adjusted for sex and age by user category. The prevalences of dietary supplement use of each major category of users and of the main sub-categories of users among males and females were compared by the chi-squared test by user category. Energy and nutrient intake from “all” dietary supplements among “regular users” (by sex), and major categories of dietary supplements, including (1) vitamin, (2) mineral, (6) drink type, and (8) others among “regular users” were expressed as percentiles, maximum values, and number of “excess users”. All the statistical analyses were performed using the Statistical Analysis System, release 8.2.^20^ Differences with p value less than 0.05 were considered significant.

## RESULTS

The prevalence of “all” dietary supplement users in each user category and sociodemographic and lifestyle characteristics by user categories are shown in Table 2. In this study, 55 % of males and 61 % of females consumed some kind of dietary supplement (“all” dietary supplements) in the previous year. Among these subjects, females were more likely to take dietary supplements than males (p<0.01). “Seldom users” constituted about 20 % of the subjects (males: 23%, females: 19%). “Regular users” constituted about 30 % of males (“weekly users”: 14 %, “daily users”: 18 %) and 40% of females (“weekly users”: 16 %, “daily users”: 26 %). “Seldom users” were predominant among males (p<0.05) while “daily users” were predominant among females (p<0.001). The prevalence of “all” dietary supplement users in each user category varied depending on the age group (p<0.05, adjusted for sex). “Seldom users” (p<0.001) and “weekly users” (p<0.05) were prevalent among middle-aged people, while “daily users” were prevalent among older people (p<0.001). “All” dietary supplement users were subjectively less healthy than nonusers after adjustment for sex and age (“any users”: p<0.01). However, the association was influenced by the frequency of use (“seldom users”: not significant, “weekly users”: p<0.01, and “daily users”: p<0.05). When dietary supplement use was limited to use without all prescription and non-prescription medicine, subjective health status was significantly associated with the use of dietary supplements in “any users” and “weekly users” (“any users”: p<0.01, “seldom users”: p=0.57, “weekly users”: p<0.01, “daily users”: p=0.07). “All” dietary supplement users were more careful of maintaining appropriate weight than nonusers in the “any users” category (p<0.05); however, the associations of “all” dietary supplements with other characteristics were not significant (i.e., smoking, education, marriage status, BMI, energy intake from food, alcohol intake, etc.) in all user categories.

**Table 2.** Prevalence of “all” dietary supplement users in each user category and sociodemographic and lifestyle characteristics by user categories.

		n	User category (%)^†^			

			Any^‡^	Seldom^§^	Weekly^∥^	Daily^¶^
Sex	Males	1,152	55	23	14	18
	Females	1,107	61**	19*	16	26***

Age (year)	40-49	534	65	33	17	14
	50-59	580	55	23	15	17
	60-69	562	55	17	15	24
	70-	583	57*	11***	13*	33***

Smoking	Never	1,268	61	20	15	25
	Past	524	55	21	13	21
	Current	462	54	22	16	17

Subjective health status	Excellent/Good	573	55	26	12	18
	Usual	1,433	58	19	16	23
	Bad/Very bad	244	66**	20	18**	28*

Total sum of family annual income, million yen	-4.49	668	57	15	14	28
	4.50-9.99	1,012	57	24	14	20
	10.00-	513	61	24	18	19

Education	Less than high school	671	58	15	14	28
	High school or equivalent	923	57	20	16	22
	More than high school	655	60	28	15	17

Marriage status	Unmarried	58	50	24	14	12
	Married	1,944	57	21	15	22
	Separated/Divorced	51	67	31	20	16
	Widowed	202	63	13	17	33

Body mass index (kg/m^2^)	<18.5	123	56	16	11	29
	18.5-24.9	1,588	59	21	16	22
	25.0-	547	56	21	14	21

Care of maintaining appropriate weight	Yes	1,375	60	20	16	24
	No	876	55*	22	14	20

Energy intake (kcal/day)^††^	-1500	201	58	19	13	26
	1500-1999	926	60	19	15	26
	2000-2499	759	56	22	15	20
	2500-	225	58	27	16	15

Energy intake from fat (%)^††^	<20	203	59	15	16	28
	20-24	639	56	19	14	24
	25-29	792	58	22	15	21
	30-	477	61	25	15	21

Total alcohol intake (g etanol/day)^††^	<10	1,500	60	20	16	24
	10-19	265	56	21	12	23
	20-29	139	52	24	13	15
	30-	207	51	23	12	16

Participants using any dietary supplements were defined as any dietary supplement users during the previous year.

† : Dietary supplement users were categorized into three user groups:

Seldom; seldom users those who reported any dietary supplement use once a year or more but less than once a week for the past 12 months.

Weekly; weekly uses those who reported any dietary supplement use once a week or more but less than once a day for the past 12 months.

Daily; daily users those who reported any dietary supplement use once a day or more for the past 12 months.

‡ : n=1,306 (628 males and 678 females)

§ : n=470 (260 males and 210 females)

∥ : n=335 (158 males and 177 females)

¶ : n=501 (210 males and 291 females)

*p<0.05, **p<0.01, ***p<0.001: Sex distribution was tested by chi-squared test. Age distribution was tested by Cocran-Mantel-Haenszel chi-squared test adjusted for sex. Other variables were tested by Cocran-Mantel-Haenszel chi-squared test adjusted for sex and age

†† : Intake was settled using 3-day diet record.

The prevalence of dietary supplement users by major category and sub-category by user categories are shown in Table 3. Among major categories of dietary supplements, the most widely consumed dietary supplement was drink type (27.0%), the second was vitamin (23.1%), the third was “others” (18.3%) and the fourth was medicine (12.0%) in males. On the other hand, the most widely consumed dietary supplement was vitamin (30.2%), the second was “others” (26.9%), the third was drink type (24.8%), and the fourth was medicine (9.7%) in females. The prevalence of vitamin, “others”, and mineral dietary supplement use in females was significantly higher than that in males; however, drink type dietary supplement use in males was significantly higher than that in females in “any users”. The prevalence of amino acid, fatty acid, and dietary fiber use was only about 1 % or less in “any users”.

**Table 3.** Prevalence of dietary supplement users by major category and sub-category by user category (%) (1,152 males and 1,107 females)

Category	Sub-category		User category^†^							

			Any		Seldom		Weekly		Daily	
			
			Males	Females	Males	Females	Males	Females	Males	Females
1. Vitamin			23.1	30.2*	6.2	6.8	6.9	7.2	10.0	16.2*
	Multivitamin		14.6	15.5	4.4	4.4	5.4	4.4	4.8	6.6
	Vitamin C		4.7	8.0*	1.1	1.4	1.5	2.1	2.1	4.6*
	Vitamin E		4.0	6.8*	0.7	1.3	0.5	1.0	2.8	4.5*
	Vitamin B_2_		2.0	2.8	0.8	1.1	0.4	0.5	0.9	1.2
	Vitamin B_12_		2.3	2.4	0.6	0.6	0	0.4*	1.7	1.4
	Vitamin D		0.3	2.8*	0	0.1	0	0.5*	0.3	2.2*
	Vitamin A		0.4	1.1*	0.1	0.2	0.1	0	0.2	0.9*
	Vitamin B_1_		0.5	0.8	0.2	0.1	0.2	0.2	0.2	0.5
	Pantothenic acid		0.2	0.8*	0	0.1	0.1	0.1	0.1	0.6*
	Vitamin B_6_		0.4	0.3	0.1	0	0	0	0.3	0.3
	Vitamin K		0.1	0.5	0	0	0	0	0.1	0.5
	Folate		0.1	0	0	0	0	0	0.1	0

2. Mineral			2.7	7.6*	0.8	1.4	0.4	2.1*	1.5	4.2*
	Calcium		1.7	5.2*	0.4	0.6	0.3	1.3*	1.0	3.3*
	Iron		0.2	2.4*	0.2	0.6	0	1.0*	0	0.7*
	Magnesium		0.4	0.5	0.1	0.2	0.1	0	0.2	0.3
	Other minerals		0.5	0.5	0.2	0.1	0.1	0	0.3	0.4

3. Fatty acid			1.0	1.2	0.1	0.3	0.1	0.2	0.7	0.8

4. Amino acid			1.1	1.5	0.1	0.4	0.4	0*	0.6	1.2

5. Dietary fiber			0.1	0.5	0	0.1	0	0	0.1	0.5

6. Drink type			27.0	24.8*	17.5	14.0*	7.4	8.0	2.2	2.9

7. Medicine			12.0	9.7	10.0	8.2	1.6	0.9	0.4	0.5

8. Others			18.3	26.9*	3.0	4.6*	4.1	6.0*	11.3	16.4*

† : Dietary supplement users were categorized into three user groups:

Seldom; seldom users those who reported any dietary supplement use once a year or more but less than once a week for the past 12 months.

Weekly; weekly uses those who reported any dietary supplement use once a week or more but less than once a day for the past 12 months.

Daily; daily users those who reported any dietary supplement use once a day or more for the past 12 months.

Any; combined three groups.

* : p<0.05 by Chi square test

No subject used niacin or biotin sub-category dietary supplement.

About a half of vitamin, “others”, and mineral users consumed their respective supplements daily, whereas 60 % of drink type dietary supplement users and most medicine users consumed these supplements less frequently than once a week.

With regard to the prevalence in the sub-category of vitamin users, the prevalence of multivitamin was the highest, the second highest was vitamin C, and the third highest was vitamin E for both sexes. Calcium was the most popular nutrient in the mineral sub-category for both sexes.

Energy and nutrient intake from “all” dietary supplements among “regular users” are shown in Table 4. Median values of energy, macronutrients, minerals, and some fat-soluble vitamins (vitamin A, vitamin D, vitamin E, and vitamin K) intake from “all” dietary supplements were very few in both sexes. On the other hand, 90th percentile value of vitamin E, vitamin B group, vitamin C, and niacin intake exceeded respective nutrient intake from diet shown in the National Nutrition Survey; i.e. about 10 % or more of dietary supplement users took large amount of such nutrient from dietary supplement. “Excess users” existed for iron, magnesium (only the 6th Ed.), vitamin A, vitamin K (only the 6th Ed.), vitamin B_6_, and niacin (only the 6th Ed.).

**Table 4.** Energy and nutrient intake per day from “all” dietary supplements among “regular users”.

Nutrient	National Nutrition Survey*	Males (n=361)								Females (n=446)								
	
		Median	90th per- centile	95th per- centile	Max.	Tolerable upper intake level^†^		Excess Users^‡^		Median	90th per- centile	95th per- centile	Max.	Tolerable upper intake level^†^			Excess Users^‡^	
			
						6th edition	2005 edition	6th edition	2005 edition					6th edition	2005 edition	6th edition		2005 edition
Energy (kcal)	1930	0	16	30	363	-	-	-	-	0	30	60	237	-	-	-		-
Protein (g)	72.2	0	1	2	80	-	-	-	-	0	1	2	35	-	-	-		-
Fat (g)	54.4	0	trace^§^	1	20	-	-	-	-	0	1	1	17	-	-	-		-
Carbohydrate (g)	271.2	0	trace^§^	1	21	-	-	-	-	0	trace^§^	1	15	-	-	-		-
Calcium (mg)	546	0	126	256	1320	2500 (40-69 y.o.)	2300	0	0	0	226	400	2123	2500 (40-69 y.o.)	2300	0		0
						- (70+ y.o.)								- (70+ y.o.)				
Iron (mg)	8.1	0	trace^§^	2	129	40	55 (40-49 y.o.)	1	1	0	1	5	93	40	40 (40-49 y.o.)	1		1
							50 (50-69 y.o.)								45 (50-69 y.o.)			
							45 (70+ y.o.)								40 (70+ y.o.)			
Magnesium (mg)	259	0	9	30	808	700 (40-49 y.o.)	-	1	-	0	7	48	906	700 (40-49 y.o.)	-	1		-
						650 (50+ y.o.)								650 (50+ y.o.)				
Vitamin A (IU)	3130	0	1200	2900	10200	5000	10000	8	4	0	800	1500	11000	5000	10000	12		4
Vitamin D (IU)	328	0	26	120	726	2000	2000	0	0	0	40	140	678	2000	2000	0		0
Vitamin E (mg)	8.2	0	91	198	483	600	800 (40-69 y.o.)	0	0	0	112	210	483	600	700 (40-69 y.o.)	0		0
							700 (70+ y.o.)								600 (70+ y.o.)			
Vitamin K (*μ*g)	260	0	0	0	30000	30000	-	1	-	0	0	4	45000	30000	-	6		-
Vitamin B1 (mg)	0.87	2	38	55	280	-	-	-	-	1	43	72	144	-	-	-		-
Vitamin B2 (mg)	1.21	1	6	10	68	-	-	-	-	1	8	16	64	-	-	-		-
Vitamin B6 (mg)	1.17	1	16	41	185	100	60	3	9	1	30	66	106	100	60	3		26
Vitamin B12 (*μ*g)	7.4	0	250	1000	2340	-	-	-	-	0	500	1044	1566	-	-	-		-
Niacin (mg)	14.8	2	34	43	128	30	300^∥^	41	0	0	26	44	140	30	300^∥^	43		0
Vitamin C (mg)	101	0	210	668	6482	-	-	-	-	0	500	1100	4400	-	-	-		-

‘Weekly users” plus “daily users” were defined as “regular users”.

Seven males and 22 females were excluded from the analysis because they reported uncertainty about the information on dietary supplement intake.

Some products in which nutrient content was not described were excluded when we developed the database and we did not calculate nutrient intake from these products.

* : Results from the National Nutrition Survey in Japan, 2002 (mean of the total).

† : Tolerable upper intake level of adults in 6th edition or 2005 edition of Nutrient-Based Dietary Reference Intakes in Japan.

- : Tolerable upper intake level of adults in 6th edition or 2005 edition of Nutrient-Based Dietary Reference Intakes in Japan was not shown.

‡ : Number of participants who daily consumed some nutrients at more than the tolerable upper intake level in the 6th Edition or 2005 Edition of Nutrient-Based Dietary Reference Intakes (DRIs) in Japan.

§ : Below display limit

∥ : The amount of mg of nicotinic acid amide was used.

Energy and nutrient intake from dietary supplement by major category among “regular users” is shown in Table 5. Individuals with intake of some nutrients at the 90th percentile value were larger amount than that from diet by the National Nutrition Survey (vitamin category: vitamin E, vitamin B group, niacin, and vitamin C; Mineral category: calcium; Drink type category: vitamin B_1_, vitamin B_2_, vitamin B_6_, and niacin; “other” category: vitamin E and vitamin B group). “Excess users” existed in vitamin category (vitamin A, vitamin K, vitamin B_6_, and niacin), in the mineral category (magnesium), in drink type (niacin), and in the “others” category (iron, vitamin A, vitamin B_6_, and niacin). In the other major categories, there were no “excess users” for any nutrients.

**Table 5.** Energy and nutrient intake per day from dietary supplements by major category among “regular users”.

Nutrient	1. Vitamin^†^						2. Mineral^‡^						6. Drink type^§^						8. Others^∥^					
			
	Median	90th per- centile	95th per- centile	Max	Excess Users*		Median	90th per- centile	95th per- centile	Max	Excess Users*		Median	90th per- centile	95th per- centile	Max	Excess Users*		Median	90th per- centile	95th per- centile	Max	Excess Users*	
			
					6th edition	2005 edition					6th edition	2005 edition					6th edition	2005 edition					6th edition	2005 edition
Energy (kcal)	0	4	12	80	-	-	0	8	10	57	-	-	0	16	30	237	-	-	5	27	54	145	-	-
Protein (g)	0	0	0	2	-	-	0	0	0	1	-	-	0	0	trace^¶^	28	-	-	0	2	3	36	-	-
Fat (g)	0	0	0	2	-	-	0	0	0	trace^¶^	-	-	0	0	0	0	-	-	0	1	3	20	-	-
Carbohydrate (g)	0	0	0	9	-	-	0	0	1	2	-	-	0	0	0	13	-	-	0	1	2	21	-	-
Calcium (mg)	0	7	130	1040	0	0	126	600	920	1833	0	0	0	0	9	54	0	0	0	106	320	1200	0	0
Iron (mg)	0	0	0	12	0	0	0	4	5	10	0	0	0	0	0	4	0	0	0	4	6	129	2	2
Magnesium (mg)	0	0	0	36	0	-	0	125	300	906	1	-	0	0	0	42	0	-	0	20	60	205	0	-
Vitamin A (IU)	0	1,000	2400	8000	3	0	0	0	0	200	0	0	0	0	0	0	0	0	0	1,000	3,600	11000	12	8
Vitamin D (IU)	0	30	40	600	0	0	0	80	159	396	0	0	0	0	0	0	0	0	0	40	200	726	0	0
Vitamin E (mg)	2	182	285	483	0	0	0	0	0	10	0	0	0	0	trace^¶^	27	0	0	0	30	100	280	0	0
Vitamin K (*μ*g)	0	0	0	45000	7	-	0	6	8	66	0	-	0	0	0	11	0	-	0	4	48	400	0	-
Vitamin B_1_ (mg)	2	58	78	280	-	-	0	0	0	6	-	-	1	5	6	20	-	-	0	10	20	68	-	-
Vitamin B_2_ (mg)	trace^¶^	8	12	64	-	-	0	0	trace^¶^	6	-	-	1	4	5	10	-	-	0	3	14	68	-	-
Vitamin B_6_ (mg)	1	48	66	185	5	34	0	0	0	4	0	0	1	5	6	25	0	0	0	2	10	61	0	1
Vitamin B_12_ (*μ*g)	0	1008	1500	2340	-	-	0	0	0	4	-	-	0	0	0	10	-	-	0	9	30	62	-	-
Niacin (mg)	0	39	60	140	62	0	0	0	0	15	0	0	4	20	20	100	6	0	0	7	18	128	10	0
Vitamin C (mg)	0	700	1336	4400	-	-	0	40	50	1000	-	-	0	44	50	2500	-	-	0	90	360	1620	-	-

‘Weekly users” plus “daily users” were defined as “regular users”.

Because there were few “regular users”, 3.fatty acid, 4.amino acid, 5.dietary fiber, and 8.medicine were omitted.

Seven males and 22 females were excluded from the analysis because they reported uncertainty about the information on dietary supplement intake.

Some products for which nutrient content was not described were excluded when we developed the database and we did not calculate nutrient intake from these products.

* : Number of participants who daily consumed some nutrients at more than the tolerable upper intake level (UL) in the 6th Edition or 2005 Edition of Nutrient-Based Dietary Reference Intakes (DRIs) in Japan.

† : n=451 (191 males and 260 females)

‡ : n=85 (21 males and 64 females)

§ : n=232 (110 males and 122 females)

∥ : n=302 (129 males and 173 females)

- : Tolerable upper intake limit of adults in 6th edition or 2005 edition of Nutrient-Based Dietary Reference Intakes in Japan was not shown.

¶ : Below display limit

According to the 6th Ed. UL, 20 people were “excess users” of vitamin A among “all” dietary supplement users (Table 4), 12 among the “others” category and 3 among vitamin category (Table 5). This indicates that 5 people consumed excess doses of vitamin A from more than one dietary supplement category. Some people consumed excess doses of magnesium (one participant), vitamin B_6_ (one participant), and niacin (six participants) from more than one dietary supplement category.

## DISCUSSION

We conducted this study to evaluate the information on dietary supplement use and nutrient intake from these products in a random sample of a community-living population. Dietary supplements were used by more than half of the respondents in the previous year. The intake of some minerals and vitamins from these products were equal or more than the daily intake from food in the National Nutrition Survey.^16^ Some users were found to take excess doses of minerals or vitamins from these products.

The prevalence of “all” dietary supplement use among “any users” in the previous year in our study was more than 50 % among both sexes. This is relatively high in comparison to those reported from Japan,^21^^,^^23^ but it is almost the same as the prevalence found in studies that were conducted in the US.^2^^,^^20^^,^^22^ However, differences in the definition of dietary supplements, dietary supplement users, duration of the study period (e.g., not specified or previous one year), and survey method (e.g., questionnaire only or including interview) among these studies make direct comparisons difficult.

In the National Nutrition Survey in Japan (J-NNS) in 2001,^23^ dietary supplements were defined only as products which contained vitamins and minerals, and a concrete study period was not specified. Under this condition, 17.0 % of males and 23.6 % of females reported usual use of dietary supplements. In the subgroup of the Japan Public Health Center-based Prospective Study on Cancer and Cardiovascular Disease Cohort II,^21^ dietary supplement was investigated in a questionnaire survey. In this study, dietary supplement was classified into multivitamins, beta-carotene, vitamin C, vitamin E, and others, and dietary supplement users were defined as subjects who used a dietary supplement one or more times a week for a year or longer. In this situation, the prevalence of dietary supplement use was 10.9 %.

Survey method (e.g., questionnaire only or including interview) may be another methodological factor to affect the prevalence of the dietary supplement intake. Third National Health and Nutrition Examination Survey in 1999-2000 (NHANES III)^22^ in the US was conducted by household interviews. In NHANES III, dietary supplements included non-vitamin and non-mineral products, the duration of the study period was the previous month. Under this condition, the prevalence of dietary supplement use was 52 %, and it was similar to our results. It is possible that relatively high prevalence of dietary supplement use found in NHANES III and our study may result from the use of survey methods including interview.

At present, there have been a few studies on dietary supplement assessment methodology.^5^^-^^7^^,^^11^^,^^21^^,^^24^^-^^27^ It is important to develop generally accepted assessment method in the dietary supplement study to make direct comparison.

We clarified the characteristics of dietary supplement users for the first time in Japan. Many studies conducted in the US and European countries reported that dietary supplement use was related to many aspects of appropriate lifestyles and a high health status.^6^^,^^9^^,^^13^^,^^28^^-^^33^ In contrast, dietary supplement users in this study were likely to feel less healthy than nonusers. Dietary supplement users might have been more careful of maintaining an appropriate weight than nonusers, whereas other characteristics (i.e., smoking, education, marriage status, BMI, energy intake from food, and alcohol intake) were not significantly associated with dietary supplement use or nonuse in this study. The characteristics of dietary supplement users in our study might have been different from the characteristics of dietary supplement users in other countries. Such characteristics may depend on sex, age, and ethnicity.^7^^-^^9^^,^^28^ Furthermore, some characteristics were different between frequencies of use of dietary supplements. For example, “seldom users” were prevalent among middle-aged subjects and were more likely to be males, whereas “daily users” were prevalent among older people and more likely to be females in our study. The association between dietary supplement use and other characteristics may be affected by the frequency of use of dietary supplements.

Multivitamin, vitamin C, and vitamin E were the popular dietary supplements in the vitamin category, and calcium was the most popular dietary supplement in the mineral category in our study. In the US, approximately 40% of subjects was reported to be users of some vitamin or mineral supplements in the NHANES III.^8^ About 40 to 80% of adults was reported to be users of some vitamin or mineral supplements. Multivitamins were the most popular dietary supplements, and vitamin C, vitamin E, vitamin A, and calcium were commonly used in vitamins and mineral supplements.^8^^,^^9^^,^^13^^,^^28^^,^^29^^,^^31^^,^^34^^-^^36^ Many studies reported the prevalence of combined dietary supplement use (vitamins and mineral). The prevalence of use of each dietary supplement was not determined; however, our results (vitamin plus mineral: males 25.8%, females; 37.8%) would be broadly comparable to the results of those studies.

Schaffer et al^33^ reported that the prevalence of non-vitamin and non-mineral dietary supplement use was 32.7% (participants were the members of a large group in a model health plan, the duration of the study period was the past 12 months, and dietary supplement use was assessed by a questionnaire). The prevalence of non-vitamin and non-mineral dietary supplement users in our study (“all” - vitamin - mineral: males; 28.7%, females; 23.5%) was close to the results of Schaffer et al. Radimer et al^32^ reported that non-vitamin and non-mineral dietary supplements included many herbal supplements in NHANES III, and the term “herbal” is often used loosely, including non-plant dietary supplement (i.e., enzymes, glandular extracts, choline, and fish oils). Herbal supplements were most commonly used because they were considered “healthy or good for you”,^14^^,^^34^^,^^37^^,^^38^ and consumers may perceive plant products as more natural than manufactured medicines.^38^ Furthermore, some studies reported that herbal supplement use is accelerating, and some products might have adverse health effects.^8^^,^^32^^,^^33^^,^^38^ We could not determine the reason why some individuals chose “others” types of dietary supplements. As the prevalence of “others” types of dietary supplement use was high in our study, it will be important to estimate the prevalence of this kind of product (non-vitamin and non-mineral dietary supplements) and to clarify the health effects of these products.

In our study, the prevalence of drink type dietary supplement was high, especially in males, and more than 60 % of drink type dietary supplement users were “seldom users”. Hakura et al^14^ also reported that high prevalence of drink type dietary supplement was observed in Japan, and many of the occasional dietary supplement users took this kind of dietary supplements to maintain or recover health. However, drink type dietary supplement was not usually described in the studies reported from the Western countries.^32^^-^^34^^,^^38^ The high prevalence of drink type dietary supplement use might be one of the characteristics observed in Japan, and might be caused by broad accessibility that people can get drink type dietary supplements easily at supermarkets and convenience stores when they feel weary.

The purpose of dietary supplement use may be to compensate the shortage of nutrients from foods, but some users had excessive intake of some nutrients. The median values of Vitamin B_1_ and 90 percentile values of vitamin E, Vitamin B_2_, B_6_, B_12_, niacin, and vitamin C from dietary supplements in this study were more than that from food, according to the results of J-NNS 2002.^16^ Some dietary supplement users consumed huge amounts of nutrients from dietary supplements.

Regarding overdoses, this study had two important findings. The first was that overdoses sometimes occurred for non-target nutrients from dietary supplements, when the primary nutrient in the dietary supplement was defined as the target nutrient. For example, according to the 6th Ed. UL, only three persons took an excess dose of vitamin A among vitamin supplement users, whereas 12 people consumed an excess dose of vitamin A among the “others” type of supplement users (Table 5). The second was that overdose sometimes occurred in users of “multiple” dietary supplements. In this study, according to the 6th Ed. UL, five people consumed an excess dose of vitamin A from “multiple” dietary supplements which belonged to different categories.

Stewart et al^31^ reported that there was a wide range of intake of vitamins from dietary supplements. Subjects who took more than 10 times the Recommended Dietary Allowances (RDAs) in the US were observed for vitamin B group, vitamin C, vitamin E, niacin, and pantothenic acid intakes. Other studies reported that some dietary supplement users consumed excess doses of some nutrients as compared to the RDAs.^6^^-^^9^^,^^39^^,^^40^ Rock et al^2^ noted that a few women consumed potentially toxic levels of vitamin A, vitamin B_6_, iron, and zinc from dietary supplements. People need to be aware that excessive use of some dietary supplements may produce undesirable health effects.^41^^,^^42^ Because we did not include fortified foods and modified foods among dietary supplements in this study, nutrient intake from those foods was not included the estimation of total nutrient intake. We are apprehensive that excessive levels of nutrient intake could be more common people with in a combination of fortified foods, modified foods and dietary supplement use.

The main strength of this study is the development of the nutrient content database of more than 900 dietary supplements, and the use of this database to calculate nutrient intake from these products for more than 2,000 middle-aged and older people. Although our database of dietary supplements is extensive, a lack of information on some dietary supplements still exists. Information on the nutrient content of some products available in the marketplace had not been obtained even by the producer and/or was difficult to get,^6^^,^^7^^,^^43^^-^^45^ because dietary supplements except for medicines are not required to show their nutrient contents.

## APPENDIX

We succeeded in constructing the database of more then 1500 dietary supplement products in April 2006. The database has been regularly updated according to the study. We will make latest dietary supplement database generally available, but for non-profit use only, in the internet website (http://www.nils.go.jp/department/ep/index-j.html) of our institute, without a need for permission. The authors, however, request that this article be cited when a study in which the data, or even a part of it, were used is published or open to the public. We expect that this database will be useful for the prevention of excess intake of dietary supplements and contribute to the development of research on nutritional epidemiology.
